# Critical role of matrix metallopeptidase 9 in postoperative cognitive dysfunction and age-dependent cognitive decline

**DOI:** 10.18632/oncotarget.15545

**Published:** 2017-02-20

**Authors:** Jiangjiang Bi, Weiran Shan, Ailin Luo, Zhiyi Zuo

**Affiliations:** ^1^ Department of Anesthesiology, University of Virginia, Charlottesville, Virginia, USA; ^2^ Department of Anesthesiology, Tongji Hospital, Huazhong University of Science and Technology, Wuhan, Hubei, China

**Keywords:** age-dependent cognitive decline, matrix metallopeptidase 9, neuroinflammation, postoperative cognitive dysfunction

## Abstract

**Background:**

Postoperative cognitive dysfunction (POCD) is a significant clinical syndrome. Neuroinflammation is an important pathological process for POCD. However, it is not clear how systemic inflammation induced by surgery on peripheral tissues or organs is transmitted into the brain. We determined whether matrix metallopeptidase 9 (MMP9), a protein that can increase blood-brain barrier permeability, is critical in this transmission. The role of MMP9 in age-dependent cognitive decline was also determined.

**Methods:**

Two-month old male C57BL/6J wild-type mice and MMP9^-/-^ mice were randomly assigned to control or surgery groups. The surgery was right carotid artery exposure under isoflurane anesthesia. Cognitive function was tested from one week after the surgery by Barnes maze and fear conditioning. Cognitive function of 2-month old C57BL/6J mice was compared with that of 18-month old mice.

**Results:**

Surgery increased the expression of interleukin 1β, interleukin 6 and ionized calcium binding adapter molecule 1, inflammation indicators, in the brain of the wild-type mice. Blood-brain barrier permeability was increased by surgery. Surgery also impaired the learning and memory of these mice. These surgical effects were absent in the MMP9^-/-^ mice. Eighteen-month old wild-type mice had poorer performance in Barnes maze and fear conditioning tests and lower MMP9 protein expression and activity than did the 2-month old mice.

**Conclusion:**

MMP9 is critical for transmission of systemic inflammation into the brain for POCD. MMP9 may also play a role in age-dependent cognitive decline.

## INTRODUCTION

Postoperative cognitive dysfunction (POCD) is a significant clinical syndrome affecting 30 to 40% patients at hospital discharge and about 10% patients 3 months after non-cardiac surgeries [1]. POCD may be associated with increased mortality and withdrawal from job market [1, 2]. Age is a risk factor for the occurrence of POCD [1]. Currently, no effective treatment for POCD has been developed for clinical use. It is necessary to understand the mechanisms/pathophysiology for POCD so that specific interventions can be designed to reduce the occurrence of POCD.

We and others have shown that neuroinflammation is a critical pathophysiological process for POCD [3, 4]. However, it is not clear how systemic inflammation induced by surgery can be transduced into the brain. Matrix metallopeptidase 9 (MMP9) is a gelatinase that can breakdown extracellular matrix [5]. MMP9 is an important enzyme that contributes to hemorrhagic transformation after ischemic stroke [6–8]. Our previous study has shown that surgery increases the expression of active MMP9 in the brain [9]. Thus, MMP9 may participate in the increased permeability of blood-brain barrier (BBB) after anesthesia and surgery. This increased permeability may allow inflammatory molecules or cells in the blood to go through the BBB into the brain parenchyma, which ultimately induces neuroinflammation.

Based on the above information, we hypothesize that MMP9 is critical for the development of cognitive dysfunction and neuroinflammation after surgery. In this study, we used MMP9^-/-^ mice to address this hypothesis. We also compared the expression of MMP9 between young and old mouse brains to determine whether MMP9 may contribute to the age-dependent cognitive decline.

## RESULTS

### Surgery induced cognitive dysfunction in the wild-type mice but not in the MMP9^-/-^ mice

No animals died during the study. All animals assigned to the study contributed data presented in the following sections.

**Figure 1:**
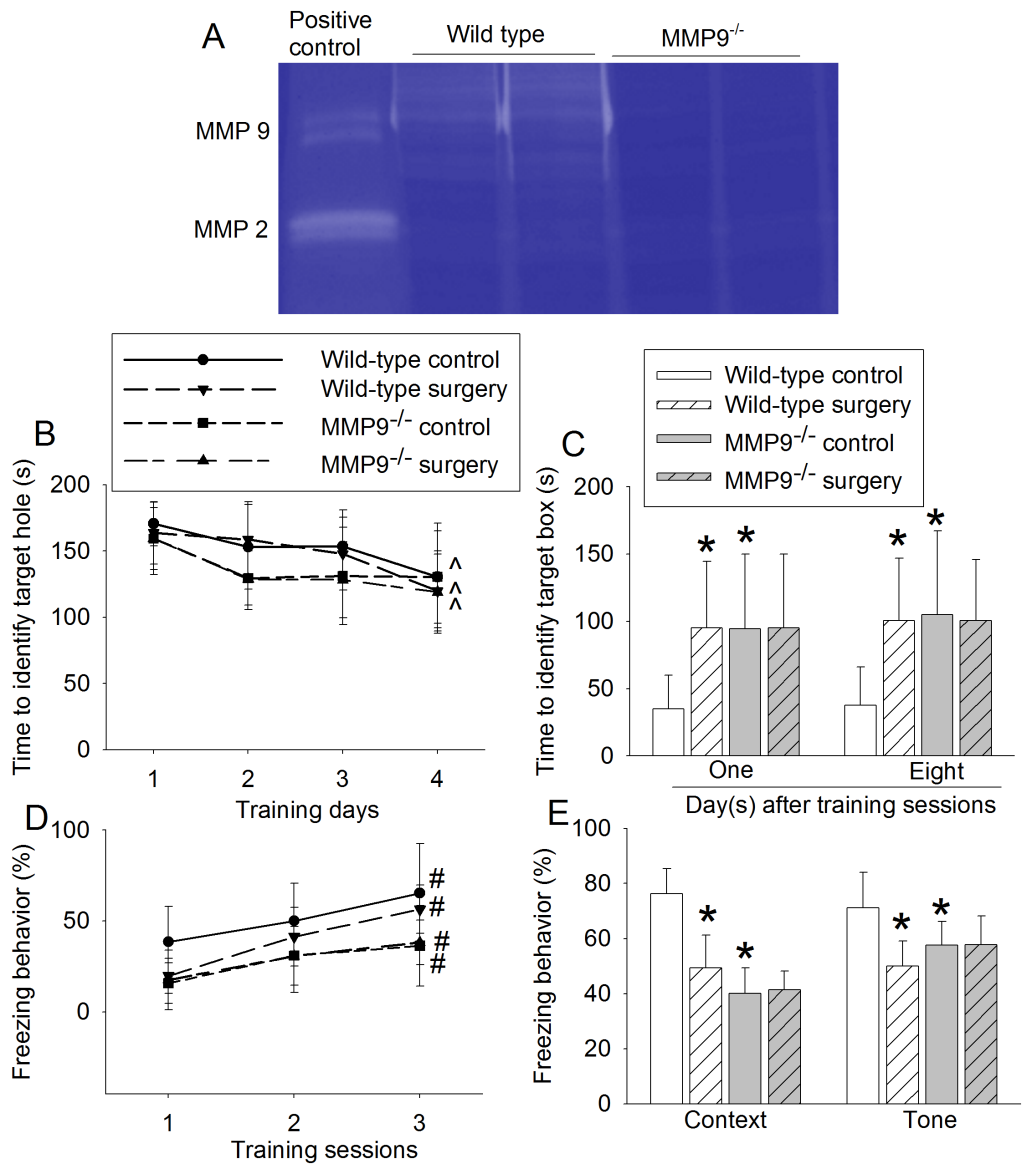
Effects of surgery on learning and memory in wild-type and MMP9^-/-^ mice **A.** representative images of MMP9 and MMP2 gelatin zymography; **B.** performance during the training phase of Barnes maze test; **C.** performance during the memory phase of Barnes maze test; **D.** performance during the training phase of fear conditioning test; and **E.** performance during context- and tone-related fear conditioning test. Results are mean ± S.D. (*n* = 10). ^ *P* < 0.05 compared with the corresponding values on day 1, * *P* < 0.05 compared with values of wild-type control mice. # *P* < 0.05 compared with the corresponding values in training session 1.

There were bands at about 85 KDa, corresponding to MMP9, in the gelatin zymographic assay with using wild-type mouse spleen. These bands did not exist in the assay with using spleen of the MMP9^-/-^ mice. There were also zymographic bands at about 62 KDa that was corresponding to MMP2 in the mouse spleen. These bands did not appear to be affected in the MMP9^-/-^ mouse tissues (Figure 1A). These results indicate that MMP9 is knocked out in the MMP9^-/-^ mice and that this knockout did not change MMP2 activity. We used spleen to measure MMP2 and MMP9 activity because we could not detect a clear band corresponding to MMP2 in the brain tissues of both wild-type and MMP9^-/-^ mice.

Wild-type mice and MMP9^-/-^ mice in the control and surgery groups took less time on the fourth training day than on the first training day to identify the target hole in the Barnes maze test, suggesting that mice in all groups improved their performance with training (Figure 1B). Surgery and MMP9 knockout were not a significant factor to affect the mouse performance in these training sessions [F(1, 18) = 0.496, *P* = 0.490; F(1, 18) = 3.465, *P* = 0.079; respectively, for surgery and MMP9 knockout factors]. However, wild-type mice in the surgery group took a longer time than did control mice to identify the target box when they were assessed one day or eight days after the training sessions in Barnes maze, suggesting that surgery induces learning and memory impairment in the wild-type mice. Interestingly, surgery did not affect the time for MMP9^-/-^ mice to identify the target box at one day or eight days after training sessions. However, the MMP9^-/-^ control mice took longer than wild-type control mice to identify the target box (Figure 1C).

Similar to the situation in Barnes maze test, animals in all four groups had more freezing behavior with increased training in the fear conditioning test (Figure 1D). MMP9 knockout was a significant factor to decrease the freezing behavior during these training sessions [F(1,18) = 8.585, *P* = 0.009]. Wild-type mice in the surgical group had less freezing behavior than did control mice in the context- and tone-related fear conditioning test. Although surgery did not change the freezing behavior in the MMP9^-/-^ mice, MMP9^-/-^ control mice had less freezing behavior than wild-type control mice in the context- and tone-related fear conditioning test (Figure 1E).

### Surgery induced neuroinflammation and increased BBB permeability in the wild-type mice but not in the MMP9^-/-^ mice

**Figure 2:**
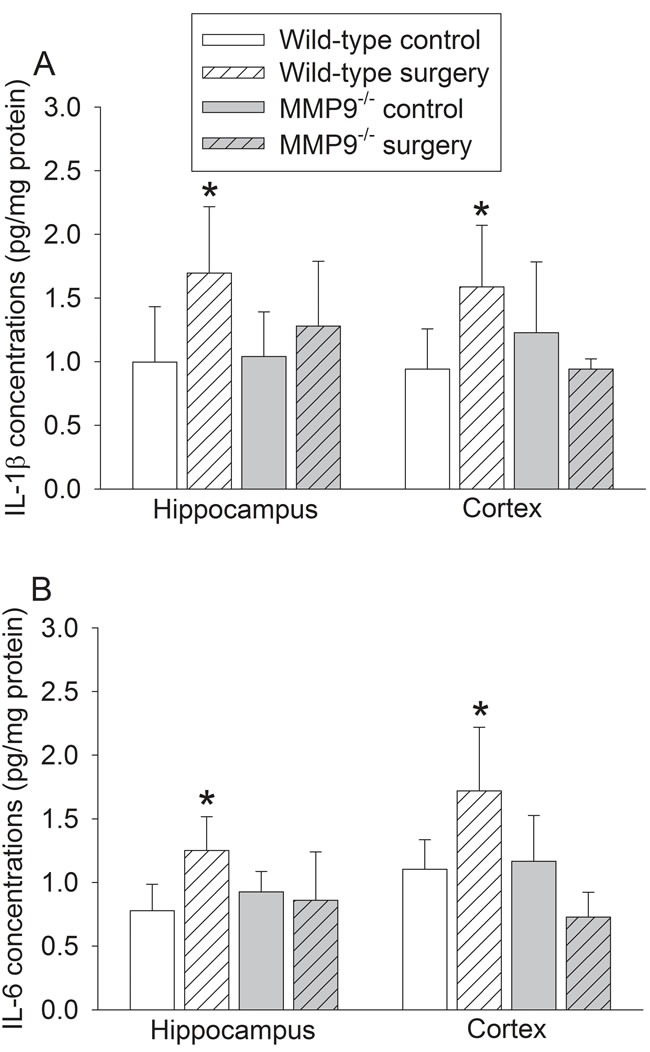
Effects of surgery on proinflammatory cytokine expression in wild-type and MMP9 **^-/-^** mice. **A.** IL-1β; **B.** IL-6. Results are mean ± S.D. (*n* = 6). * *P* < 0.05 compared with values of wild-type control mice.

**Figure 3:**
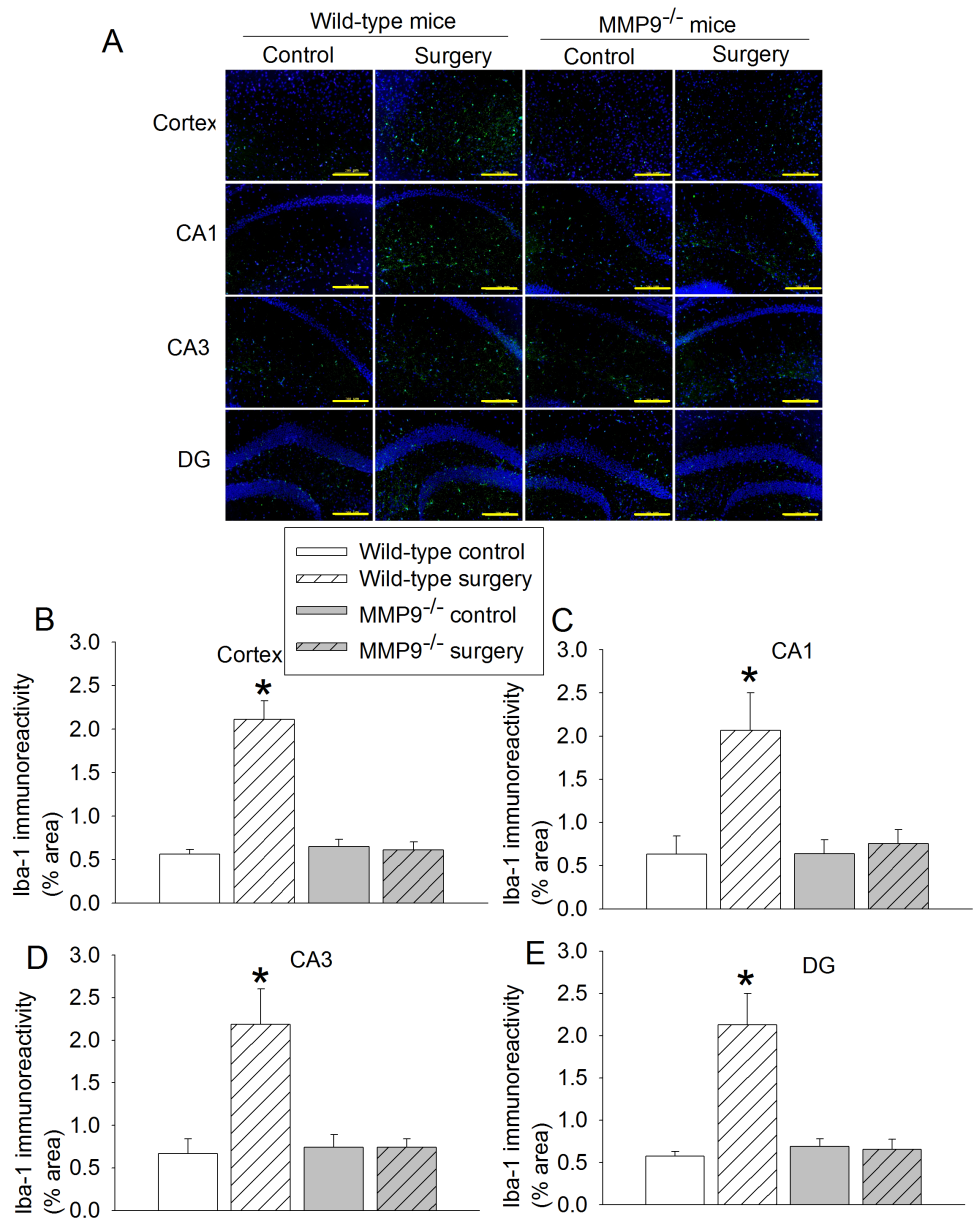
Effects of surgery on Iba-1 expression in wild-type and MMP9^-/-^ mice **A.** representative images of Iba-1 (green) and Hoechst 33342 (blue) staining, scale bar in each panel = 200 µm; **B.** to **E.** quantification of Iba-1 immunoreactivity in cerebral cortex, CA1, CA3 and dental gyrus (DG). Results are mean ± S.D. (*n* = 6). * *P* < 0.05 compared with values of wild-type control mice.

**Figure 4:**
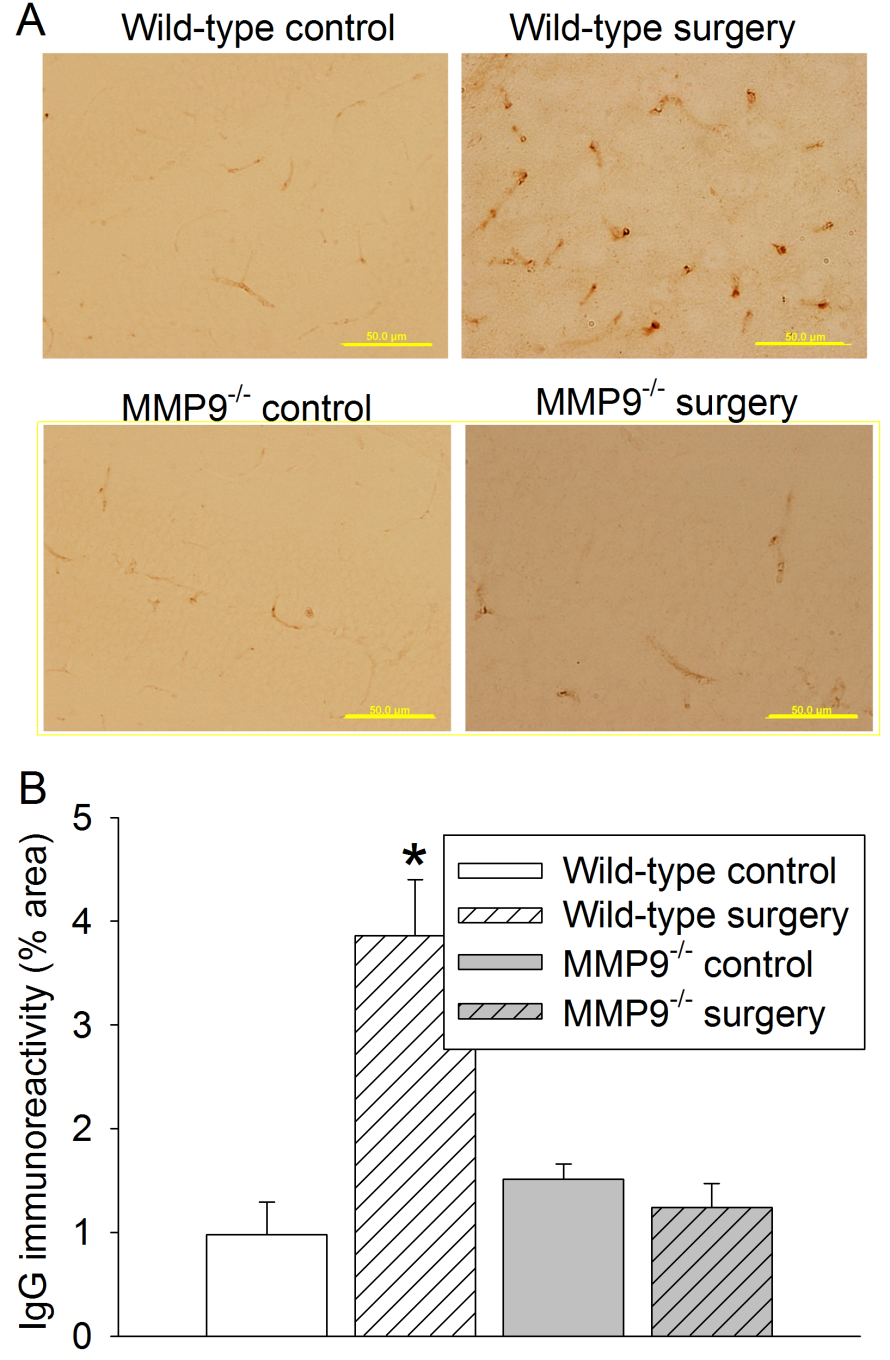
Effects of surgery on permeability of IgG into brain tissues in wild-type and MMP9 **^-/-^** mice. **A.** representative images of IgG staining (brown) in CA3, scale bar in each panel = 50 µm; **B.** quantification of IgG immunoreactivity in CA3. Results are mean ± S.D. (*n* = 6). * *P* < 0.05 compared with values of wild-type control mice.

Surgery significantly increased the expression of interleukin (IL)-1β, IL-6 and ionized calcium binding adapter molecule 1 (Iba-1) in the hippocampus and cerebral cortex of wild-type mice (Figures 2 - 3). Surgery also increased the amount of IgG in the brain tissues (Figure 4). These surgery-induced effects were not presented in the MMP9^-/-^ mice (Figures 2 - 4).

### Older mice had poorer learning and memory and had less MMP9 in the brain than did younger mice

**Figure 5:**
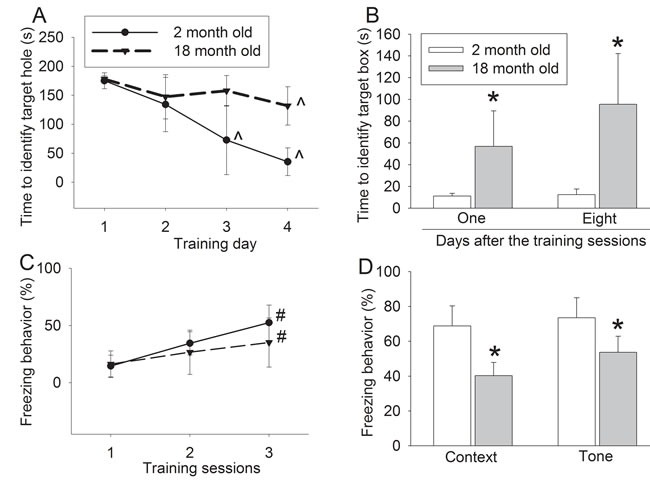
Effects of age on learning and memory in wild-type mice **A.** performance during the training phase of Barnes maze test; **B.** performance during the memory phase of Barnes maze test; **C.** performance during the training phase of fear conditioning test; and **D.** performance during context- and tone-related fear conditioning test. Results are mean ± S.D. (*n* = 8). ^ *P* < 0.05 compared with the corresponding values on day 1, * *P* < 0.05 compared with values of 2-month old mice. # *P* < 0.05 compared with the corresponding values in training session 1.

Although both 2- and 18-month old mice improved their performance in the training sessions of Barnes maze (Figure 5A), age was a significant factor to increase the time to identify the target box in the training sessions [F(1, 14) = 22.066, *P* < 0.001]. Eighteen-month old mice also took a longer time to identify the target box at one day and eight days after the training sessions than did two-month old mice (Figure 5B). Similarly, age trended to be a significant factor to affect the freezing behavior in the training sessions of fear conditioning test [F(1, 14) = 1.327, *P* = 0.269] (Figure 5C). Eighteen-month old mice had less freezing behavior in the context- and tone-related freezing behavior than two-month old mice (Figure 5D).

**Figure 6:**
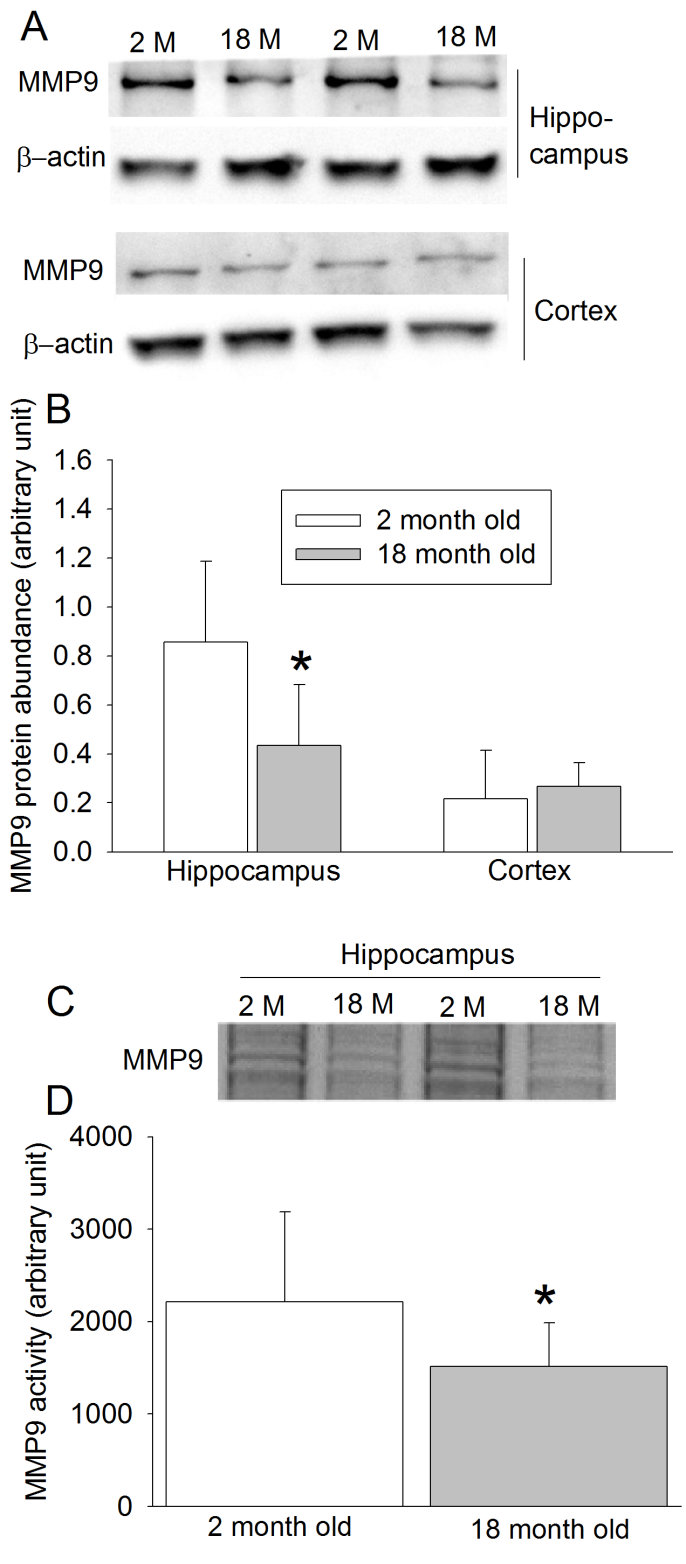
Effects of age on MMP9 protein expression and activity in wild-type mice **A.** Representative images of Western blotting; **B.** quantitative results of Western blotting; **C.** Representative images of MMP9 gelatin zymography; **D.** quantitative results of gelatin zymography. Results are mean ± S.D. (*n* = 8 for results in panels B and D). * *P* < 0.05 compared with values of 2-month old mice.

The expression of MMP9 protein in the hippocampus was decreased in the 18-month old mice (Figures 6A and 6B). Consistent with this finding, the MMP9 activity was also decreased in the elderly mice (Figures 6C and 6D). However, the expression of MMP9 in the cerebral cortex may not be changed in the elderly mice (Figures 6A and 6B).

## DISCUSSION

Our results clearly suggest a critical role of MMP9 in surgery-induced cognitive dysfunction and neuroinflammation. Consistent with our previous studies [3, 9, 10], carotid artery exposure induced learning and memory impairment as reflected by the need of increased time to identify the target box in the Barnes maze test and reduced freezing behavior in the fear conditioning test. This surgical procedure also induces neuroinflammation as indicated by increased IL-1β and IL-6, proinflammatory cytokines, and Iba-1, a microglial marker [3, 9]. These surgical effects were abolished in the MMP9^-/-^ mice, suggesting a critical role of MMP9 in these effects.

Surgery on peripheral tissues or organs can induce systemic inflammation [11]. Recent studies have shown that surgery also induces neuroinflammation and that neuroinflammation may be an important neuropathological process for POCD [3, 4, 9, 12, 13]. The transmission of systemic inflammation into the brain may involve the translocation of proinflammatory cytokines and/or cells, such as monocytes, from the blood into the brain [14] and, therefore, require the increase of BBB permeability [15]. We have shown that the active form of MMP9 protein in the brain is increased in rodents after carotid artery exposure [9]. The expression of MMP9 in the brain is increased after an open tibial fracture and fixation surgery in the elderly rats [16]. MMP9 is known to break down extracellular matrix, which can disrupt BBB [17]. Thus, MMP9 may help the transmission of systemic inflammation into the brain. Consistent with this idea, we showed that surgery increased BBB permeability and this increase was abolished in the MMP9^-/-^ mice. MMP2 and MMP9 are the major gelatinases. Our results confirmed that MMP9 was knocked out in the MMP9^-/-^ mice. MMP2 activity did not appear to have a compensatory change in these mice. Thus, surgery-induced systemic inflammation may increase the MMP9 activity in the brain vascular structures, which increases BBB permeability to allow proinflammatory mediators/cells to get into the brain to induce neuroinflammation and impairment of learning and memory. Consistent with our findings, a prolonged exposure (4 h) to isoflurane increased BBB permeability and induced learning and memory impairment in elderly rats [18]. Of note, we did not include a group of anesthesia only in our study because anesthesia is often used in patients who are having surgery.

In addition to increasing BBB permeability, MMP9 also can directly participate in inflammation by activating proinflammatory cytokines and helping inflammatory cells to migrate to inflammatory regions [17, 19]. These effects may further enhance the neuroinflammation after surgery. Our previous study has shown that inflammatory cytokines inhibit the translocation of GluR1, an α-amino-3-hydroxy-5-methyl-4-isoxazolepropionic acid receptor subunit, to the plasma membrane of neurons [20], a process that is a biochemical component for learning and memory. Thus, MMP9 may facilitate the effects of inflammation on neurons to induce learning and memory dysfunction.

The MMP9^-/-^ mice needed more time to identify the target box in the Barnes maze test and had less freezing behavior in the fear conditioning test than did wild-type mice. These results suggest that MMP9 knockout impairs learning and memory. This impairment is not due to neuroinflammation because MMP9^-/-^ mice did not have an increased level of IL-1β, IL-6 and Iba-1 in their brain. The involvement of MMP9 in learning and memory has been described previously. MMP9 can facilitate the synaptic plasticity and long term potentiation [19, 21, 22]. MMP9^-/-^ mice have decreased dendritic length and complexity [23]. Consistent with these results, we showed here that elderly mice had decreased MMP9 expression and activity in the hippocampus and declined learning and memory as reflected by needing longer time to identify the target box in the Barnes maze and having less freezing behavior in the fear conditioning test. These novel results suggest that MMP9 plays a role in the age-dependent decline of learning and memory. This role is consistent with the known function of MMP9 in synaptic plasticity. However, MMP9 reduction shall also inhibit neuroinflammation and disruption of BBB induced by insults, such as surgery. This inhibition shall be beneficial when insults exist as shown in this study for animals with surgery. However, MMP9 reduction under control condition may cause detrimental effects as in the elderly animals due to the loss of its physiological functions.

The lack of effects of surgery on learning and memory in the MMP9^-/-^ mice may not be due to the ceiling effects. The results of MMP9^-/-^ mice in the training sessions of fear conditioning and Barnes maze tests suggest that these mice can learn and memorize tasks. Thus, their learning and memory are not in the worst status yet.

We used IgG immunostaining to quantify the damage of BBB. Various methods including *in vivo* imaging techniques have been developed for the purpose [24, 25]. Indicators of various molecular sizes are used. IgG is much larger than many of those indicators, such as evans blue, and, therefore, may not be as sensitive as those small molecule indicators to detect BBB damage.

Our results suggest a role of MMP9 in surgery-induced BBB disruption because this disruption disappeared in the MMP9^-/-^ mice. In addition to MMPs [17, 26], many other factors, such as oxidative stress and proteases including elastase, can also disrupt BBB [27].

Of note, wild-type 2-month old animals in the control group that contributed data to Figure 5 appeared to perform better than the same type of mice that contributed data to Figure 1 in the Barns maze test after training day 3. Many factors, such as different sets of animals and subtle change in environment, can affect the performance of animals in the learning and memory test. Our results suggest that importance of assigning the same set of animals to different groups of one experiment and assessing their learning and memory at the same time.

Our results may have significant implications. First, our results suggest a critical role of MMP9 in mediating surgery-induced neuroinflammation and impairment of learning and memory. These findings identify MMP9 as a novel target for reducing POCD. Methods to temporarily inhibit MMP9 may be developed and used to reduce POCD and surgery-induced neuroinflammation. This inhibition shall be very short because MMP9 is also known to play a critical role in spine and dendritic development [19, 21–23]. In addition, MMPs including MMP9 contribute to proper wound healing [28], which is important during perioperative period. Nevertheless, we did not find impairment in the neck wound healing in the MMP9^-/-^ mice. Second, we also showed that MMP9 might contribute to the age-dependent learning and memory impairment. If this finding is confirmed, intervention may be developed to reduce this age-dependent dysfunction.

Our study has limitations. We have not determined how MMP9 in the brain may be activated. It is known that nuclear factor κB (NFκB), a transcription factor for increasing proinflammatory cytokines, can increase MMP9 [29]. It is possible that systemic inflammation increases MMP9 in the vascular structures including the microvascular structures in the brain, which then increases the permeability of BBB. Consistent with this possibility, our previous study has shown that surgery-induced increase of active MMP9 was inhibited by a NFκB inhibitor [9]. We have not measured MMP9 expression and activity in the microvessels of the brain. This measurement in mouse brain has been very difficult in our hands due to insufficient samples. We did not include elderly MMP9^-/-^ mice in the study to determine whether MMP9 plays a role in the neuroinflammation and dysfunction of learning and memory in the elderly mice. However, these elderly MMP9^-/-^ mice are very difficult to obtain. In addition, the ceiling effect of learning and memory impairment in these elderly MMP9^-/-^ mice may confront the interpretation of the results.

In summary, we have shown that MMP9 may be critical in surgery-induced neuroinflammation and impairment of learning and memory. MMP9 also may play a role in age-dependent learning and memory dysfunction.

## MATERIALS AND METHODS

### Animals

All animal protocols were approved by the Institutional Animal Care and Use Committee of the University of Virginia (Charlottesville, VA, USA). All surgical and experimental procedures were carried out in accordance with the National Institutes of Health Guide for the Care and Use of Laboratory Animals (NIH publications number 23-80) revised in 2011. All mice were kept in a vivarium room at constant temperature (23 ± 2℃) and 12 h light/dark cycle with free access to food and water. Two-month old male C57BL/6J mice were purchased from Charles River (Wilmington, MA). Eighteen-month old male C57BL/6J mice were provided by the National Institute of Aging (Bethesda, MD). A pair of MMP9^-/-^ mice with C57BL/6J gene background were obtained from the Jackson Laboratories (stock number: 007084; Bar Harbor, ME). They were bred in our vivarium.

### Animal groups

In the first experiment, 2-month old male wild-type or MMP9^-/-^ mice were randomly assigned to two groups: control group and surgery group. The surgery was right carotid artery exposure. Their learning and memory were assessed by Barnes maze and fear conditioning tests from 6 days after the surgery (*n* = 10). Separate mice with the same experimental conditions were sacrificed at 24 h after surgery. Their brains were harvested for immunohistochemistry (*n* = 6) and enzyme-linked immunosorbent assay (ELISA) (*n* = 6).

In the second experiment, 2- and 18-month old mice without any treatment or surgery were tested by Barnes maze and fear conditioning tests (*n* = 8). Separate mice were sacrificed and their brain were harvested for Western blotting (*n* = 8) and zymographic analysis ( *n* = 8).

### Anesthesia and surgery

The animals were subjected to right carotid artery exploration surgery [3, 9, 10]. Briefly, mice were anesthetized with 2% isoflurane and mechanically ventilated. A servo-controlled warming blanket (TCAT-2LV, Physitemp instruments Inc., Clifton, NJ) was used to maintain the rectal temperature at 37°C. Mouse's heart rate and pulse oxygen saturation were monitored continuously by MouseOX Murine Plus Oximeter System (Starr Life Sciences Corporation, Oakmont, PA). A 1-cm long neck incision was made in the midline after 0.25% bupivacaine was injected subcutaneously. The soft tissues were retracted to expose the trachea. A 0.5 cm long right common carotid artery was dissected out from surrounding tissues with care to avoid damage to the vagus nerve. Isoflurane anesthesia was stopped once the wound was closed with skin staple. The surgical procedure was performed under sterile conditions and lasted for 10 min.

### Barnes maze

Six days after surgery, mice were assessed by Barnes maze test. As we previously described [9], the Barnes maze is a circular platform with 20 equally spaced holes (SD Instruments, San Diego, CA). One of the holes was connected to a dark chamber that was called target box. The test started by placing mice in the center of platform. Aversive noise (85 dB) and bright light (200 W) shed on the platform were used to encourage mice to find the target box. They were trained in 4 days continuously with 3 min per trial, 2 trials per day and 15 min between each trial. Their reference memory was then tested on day 5 (short-term retention) and day 12 (long-term retention). Each mouse had one trial on each of these two days. No test was performed during the period from day 5 to day 12. The latency to find the target box during each trial was recorded with the assistance of ANY-Maze video tracking system (SD Instruments).

### Fear conditioning

Mice were assessed by fear conditioning test as we previously described [9] 24 h after the Barnes maze test. Each mouse was placed in a test chamber wiped with 70% alcohol and subjected to three tone-foot shock pairings (tone: 2000 Hz, 85 db, 30 s; foot shock: 0.7 mA, 2 s) with a 1-min interval in a dark room. The amount of freezing behavior in each of this 1-min interval was counted (freezing behavior during training sessions). The mouse was removed from the test chamber after training. Twenty hours later, the mouse was placed back to the same chamber for 6 min without receiving tone or shock stimulation. The amount of time with freezing behavior was recorded in the 6-min interval (context-related freezing behavior). The mouse was then placed in a different test chamber wiped with lemon juice 2 h later in a light room. After 3 min without any stimuli, the tone stimulus was turned on for 30 s followed by 1-min interval for three cycles (4.5 min in total). The freezing behavior in this 4.5-min interval was recorded (tone-related freezing behavior). The time of freezing behavior was counted by an observer who was blind to group assignment of animals.

### Brain tissue harvest

Mice were deeply anesthetized with isoflurane and perfused transcardially with normal saline. Brains of mice in experiment 1 were harvested 24 hours after surgery. The left hippocampus and cerebral cortex were dissected out immediately for ELISA. The right cerebral hemisphere at Bregma -2 to -5 was harvested for immunohistochemistry. In experiment 2, the hippocampus and cerebral cortex were dissected out immediately after transcardial perfusion for Western blotting and gelatin zymography.

### Western blotting

The cytoplasmic proteins were prepared as we described before [30]. Briefly, hippocampal and cortical tissues were homogenized in RIPA buffer (Sigma-Aldrich, St. Louis, MO) containing protease inhibitor cocktail (10 mg/ml aproteinin, 5 mg/ml pepstatin, 5 mg/ml leupeptin, and 1 mM phenylmethanesulfonylfluoride) and placed on ice for 30 min. The homogenates were centrifuged at 13,000 rpm for 25 min at 4°C. The supernatant was collected for Western blotting. Protein concentration was determined by BCA assay.

The primary antibodies used were the rabbit polyclonal anti-MMP9 antibody (1:200 dilution, catalogue number: sc-10737; Santa Cruz Biotechnology, Santa Cruz, CA) and rabbit polyclonal anti-β-actin antibody (1:1000 dilution, catalogue number: 4967; Cell Signaling Technology Inc., Danvers, MA). The secondary antibody was goat anti-rabbit IgG antibody conjugated with horseradish peroxidase (1:5000 dilution; Santa Cruz Biotechnology). The densities of MMP-9 protein bands were normalized to those of β-actin from the same sample.

### Gelatin zymographic assay

As we described before [30], mice were perfused with ice-cold normal saline and their brains and spleens were harvested. Hippocampal and cortical tissues or spleen were homogenized in 50 mM Tris-HCl buffer (pH 7.4) and kept on ice for 30 min. The homogenates were centrifuged at 13,000 rpm for 25 min at 4°C. The supernatant was collected for zymography for measuring MMP9 and MMP-2 activity. Sixty microgram protein in 30 µl was mixed with the same volume of sample buffer. Forty-five microgram protein per lane was loaded onto 10% polyacrylamide gels containing 0.1% gelatin and subjected to electrophoresis. The gel was then incubated in renaturing buffer for 1 h at room temperature and developing buffer overnight at 37°C. The gel was stained in dye buffer (0.5% Coomassie blue G-250 in 40% methanol and 10% acetic acid) and then de-stained. MMP-9 activity was visualized as clear bands in the gel at appropriate molecular weights. The densities of bands were analyzed by ImageQuant TL 2005 software.

### ELISA assay of cytokines

IL-1β and IL-6 levels in the hippocampus and cortex were determined with Quantikine ELISA kits (R&D Systems, Minneapolis, MN) according to the manufacturer's instructions as we described before [31, 32]. Briefly, brain tissues were homogenized on ice in 20 mM Tris-HCl buffer (pH 7.3) for 30 min. Homogenates were centrifuged at 10,000 g for 10 min at 4°C. The supernatant was ultra-centrifuged at 150,000 g for 2 h at 4°C. The supernatant was collected for ELISA. The quantity of IL-1β and IL-6 in each sample was standardized to its protein contents.

### Immunohistochemistry

BBB disruption was assessed by IgG immunostaining of the brain sections as we previously described [30]. Cerebral hemisphere at Bregma -2 to -5 was harvested, fixed in 4% paraformaldehyde in 0.1 M phosphate-buffered saline at 4°C for 24 h, and embedded in paraffin. Coronal sections at 5 µm were cut and mounted on slides. Antigen retrieval was performed in sodium citrate buffer (10 mM sodium citrate, 0.05% Tween 20, pH 6.0) for 20 min. Endogenous peroxidase was blocked with 0.3% H_2_O_2_ for 30 min. The slides were immersed in 5% normal goat serum with 1% bovine serum albumin (BSA) in Tris-buffered saline plus 0.05% triton-X 100 (TBST) for 2 h at room temperature. The sections were incubated with biotinylated goat anti-mouse IgG antibody (1:200 dilution, catalogue number: BA-9200; Vector Laboratories, Burlingame, CA) overnight at 4°C, followed by incubation with HRP-Avidin D (1:200 dilution, catalogue number: A-2004; Vector Laboratories) at room temperature for 2 h. The staining was developed with DAB kit (catalogue number: SK-4100; Vector Laboratories).

To stain Iba-1, the antigen retrieval was performed as described above. Sections were washed in Tris-buffered saline (TBS) and blocked in 5% donkey serum with 1% BSA in TBST for 2 h at room temperature. The sections were incubated with rabbit polyclonal anti-Iba-1 antibody (1:500 dilution, catalogue number: 019-19741; Wako Chemicals USA, Richmond, VA) at 4°C overnight. Sections were rinsed in TBS. The donkey anti-rabbit IgG antibody conjugated with Alexa Fluor 488 (1:200 dilution, catalogue number: A-21206; Invitrogen, Eugene, ON) was applied for 1 h at room temperature in a dark room. After washed in TBS, sections were counterstained with Hoechst 33342 (1:1000 dilution, catalogue number: 62249; Thermo Scientific, Pittsburgh, PA) for 5 min, then rinsed and mounted with Vectashield mounting medium (catalogue number: H-1000; Vector Laboratories). Images were acquired with a fluorescent microscope with a charge-coupled device camera (Olympus DP70, Olympus Corporation, Tokyo, Japan).

In all immunostaining studies, a negative control omitting the incubation with the primary antibody was included in all experiments. Quantification was performed as we described before [3, 30]. Briefly, three independent microscopic fields in each section were randomly acquired in the hippocampal CA1, CA3, dentate gyrus (DG) or cortex area (for Iba-1 staining) or hippocampal CA3 (for IgG staining), and three sections per mouse were imaged. The number of pixels per image with intensity above a predetermined threshold level was considered to be positively stained areas. This measurement was performed by using Image J 1.47n software. The degree of positive immunoreactivity was reflected by the percentage of the positively stained area in the total area of interested structure in the imaged field. All quantitative analyses were performed in a blinded fashion.

### Statistical analysis

Results are presented as mean ± S.D. Data from the training sessions of Barnes maze and fear conditioning were analyzed by a two-way repeated measures analysis of variance followed by Tukey test. The other data were tested by a one-way analysis of variance followed by Tukey test or *t*-test as appropriate. Differences were considered significant at *P* < 0.05. All statistical analyses were performed with SigmaStat (Systat Software, Inc., Point Richmond, CA).
